# Bronchoalveolar Lavage as a Diagnostic Tool in an Atypical Pulmonary Langerhans Cell Histiocytosis

**DOI:** 10.3390/diagnostics12061394

**Published:** 2022-06-04

**Authors:** Ovidiu Fira-Mladinescu, Noemi Suppini, Gheorghe-Emilian Olteanu, Corneluta Fira-Mladinescu, Daniel Traila

**Affiliations:** 1 Center for Research and Innovation in Personalised Medicine of Respiratory Diseases, XIIIth Department—Pulmonology Discipline, “Victor Babes” University of Medicine and Pharmacy Timisoara, Eftimie Murgu Sq. no. 2, 300041 Timișoara, Romania; mladinescu@umft.ro (O.F.-M.); noemi.suppini@umft.ro (N.S.); olteanu.gheorghe@umft.ro (G.-E.O.); traila.daniel@umft.ro (D.T.); 2 Center of Expertise for Rare Lung Diseases, Clinical Hospital of Infectious Diseases and Pneumophthisiology “Dr. Victor Babes” Timisoara, Gh. Adam Street no. 13, 300310 Timisoara, Romania; 3 Research Centre in Preventive Medicine, XIVth Department—Hygiene Discipline, “Victor Babes” University of Medicine and Pharmacy Timisoara, Eftimie Murgu Sq. no. 2, 300041 Timișoara, Romania

**Keywords:** multiple cystic lung disease, cytopathologic diagnosis, immunohistochemical cell blocks

## Abstract

Pulmonary Langerhans cell histiocytosis (PLCH) is an uncommon diffuse cystic lung disease that occurs almost exclusively in young adult smokers. High-resolution computed tomography of the chest allows a confident diagnosis of PLCH in typical presentation, when nodules, cavitating nodules, and cysts coexist and show a predominance for the upper and middle lung. Atypical presentations require histology for diagnosis. Histologic diagnosis rests on the demonstration of increased numbers of Langerhans cells and/or specific histological changes. PLCH is one of the few diseases in which bronchoalveolar lavage (BAL) has a high diagnostic value and can in some circumstances replace lung biopsy. We present a case of PLCH in an elderly non-smoker. Chest imaging revealed the presence of advanced interstitial lung disease with a fibrocystic pattern. BAL cellular analyses disclosed a macrophage pattern with CD1a phenotype that strongly supports the PLCH diagnosis, even in the setting of atypical clinical presentation and a lack of smoking exposure. PLCH is extremely rare in non-smokers and could represent a distinct phenotype.

**Figure 1:**
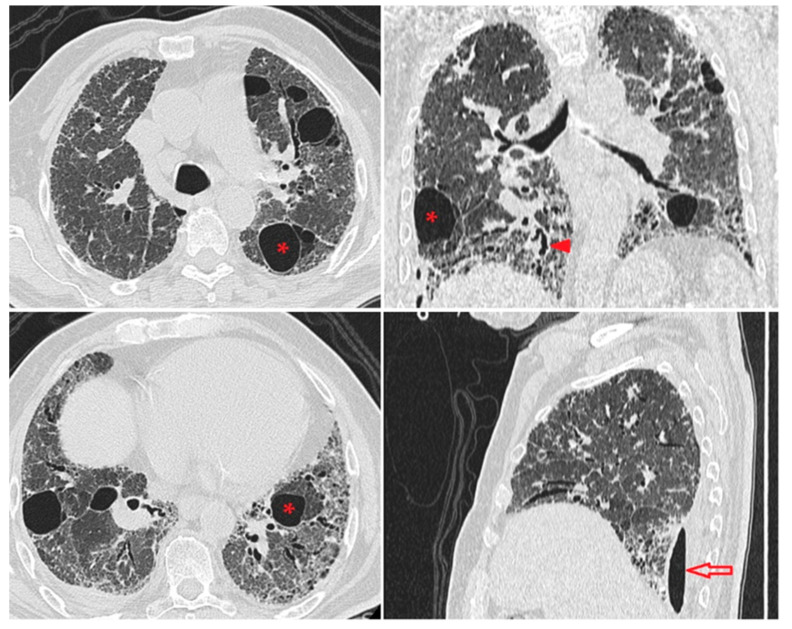
Chest high-resolution computed tomography (HRCT): diffuse “ground-glass”; subpleural reticular opacities associated with traction bronchiectasis (red arrowhead); multiple large aeric cysts in both lungs (*); loculated right lung pneumothorax (red arrow). A 70-year-old male was referred to our expert center for chronic dry cough and dyspnea on exertion (climbing stairs and walking short distances) that has slowly progressed over the last two years. He was a non-smoker with no relevant occupational exposures. His medical history revealed a previous right hydropneumothorax that was drained a year ago in a thoracic surgery department. He had multiple cardiovascular comorbidities (arterial hypertension, chronic ischemic cardiopathy, stented right coronary artery for a previous anteroseptal myocardial infarction) and type 2 diabetes treated with oral antidiabetics. The physical examination showed an overweight patient (BMI = 28.4 kg/m^2^), pallor with cyanosis at minimal exercise, grade 3 finger clubbing, lower extremity edema, basal bilateral Velcro crackles, and right pleural friction. He had oxygen saturation (SpO_2_) of 88% on room air, which increased to 92% with oxygen supplementation. Routine hematology and biochemistry investigations were unremarkable. Echocardiography revealed concentric left ventricular hypertrophy, dilation of the right ventricular cavity, stage II tricuspid regurgitation, and pulmonary hypertension (non-invasive estimation of pulmonary artery systolic pressure was 45 mmHg). Lung function tests showed a severe restrictive pattern with best forced vital capacity (FVC) of 42% pred., best forced expiratory volume in one second (FEV1) of 47% pred., and Tiffeneau–Pinelli index (FEV1/FVC) of 0.82. The chest X-ray showed reduced lung volumes with diffuse ground-glass opacification and multiple bilateral airspace opacities. Additional evaluation by chest HRCT revealed extensive bilateral fibrotic changes and cystic damage of the pulmonary parenchyma, suggestive of chronic interstitial lung disease (ILD). Clinical features, pulmonary function tests, and chest CT evaluation disclosed a longstanding undiagnosed ILD with a cystic appearance. Due to age, poor cardiologic status, and comorbidities of the patient, surgical procedures such as decortication with the re-expand of the lung and sampling of the pulmonary parenchyma could not be performed.

**Figure 2:**
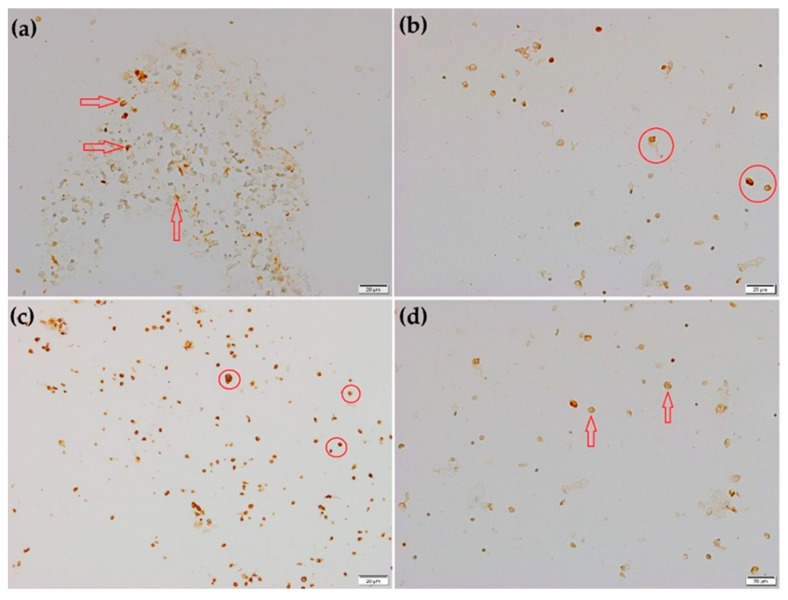
Cytoblock, immunohistochemical (IHC): (**a**) CD1a, 20×, positive membranous staining of variable intensity of Langerhans cells (red arrows); (**b**) CD4, 20×, positive cytoplasmic with membrane accentuation in lymphocytes with scattered positive macrophage cells (red circles); (**c**) CD68, 20×, intense cytoplasmic staining of macrophages, monocytes, and large lymphocytes—activated lymphocytes (red circles in order of appearance in the phrase); (**d**) S100, 20×, faint nuclear staining of Langerhans cells (red arrows). Considering the pattern on imaging of the thorax and to narrow the differential diagnosis of the chronic interstitial lung disease (ILD), a bronchoalveolar lavage (BAL) was performed. Using flexible fiberoptic bronchoscopy, 120 mL of BAL fluid with diminished translucency was collected. The collected fluid was divided into 2 equal samples: 1 sample was used for smear microscopy, and 1 sample for cytoblock evaluation. The smears with the sediment showed increased cellularity (620 × 10^3^/mL) consisting of: alveolar macrophages (89.7%) with predominantly amphophilic staining, with occasional binucleated cells, and some cells showing fine cytoplasmic granular pigment; lymphocytes (5.5%) of small and medium-size, with a regular nuclear contour and mitotically inactive; polymorphonuclear neutrophils (2.4%) with a semi-viable appearance; few squamous cells (1.2%), without atypia; ciliated bronchial epithelial cells (0.6%) predominantly in the form of dispersed cells, without atypia (image data regarding the smear evaluation not shown). Scattered eosinophilic filamentous deposits, most likely of protein origin, were noted in the background. No mast cells, plasmocytes, caliciform cells, or siderophages were identified. No microorganisms were detected. For the specific evaluation of the cells identified, the histopathological study was completed with an IHC study from formalin-fixed, paraffin-embedded tissue cell blocks. The following antibodies and clones were used in the IHC evaluation: S100 (4C4.9) anti-S100 Primary Antibody, Ventana; CD68 (KP-1), anti-CD68 Primary Antibody, Ventana; CD4 (SP35), anti-CD4 Primary Monoclonal Antibody, Ventana; CD1a (EP3622) anti-CD1a Monoclonal Antibody, Cell Marque. The pathology report showed a strong histiocytic pattern, with specific IHC staining for CD1a antibody, denoting a Langerhans cell phenotype. The analyzed cells were also positive for the monocytic antigen CD68 and faintly positive for S100. The clinical presentation, chest HRCT images (fibrotic ILD associated with multiple cysts), and characteristic BAL findings (positive CD68, CD1a phenotype, and positive S100 reaction) analyzed in a multidisciplinary discussion strongly supported the pulmonary Langerhans cell histiocytosis (PLCH) diagnosis.

PLCH is a rare disorder (<5% of ILD cases) of unknown etiology that occurs predominantly in young smokers (>90% of patients) [1]. PLHC belongs to a group of diseases distinguished by the abnormal proliferation and infiltration of different organs with Langerhans cells [2]. PLCH usually occurs as a single-system disease and is characterized by focal Langerhans cell granulomas infiltrating and destroying distal bronchioles [3,4,5,6]. The majority of patients have nonspecific respiratory symptoms (cough or dyspnea). Recurrent pneumothorax is one of the characteristic manifestations [7,8,9]. High-resolution computed tomography (HRCT) findings have been well described and consist of small and often cavitated nodules and multiple thick- or thin-walled cysts with irregular shapes, predominantly in the upper lobes of the lungs, typically sparing the costophrenic angles [10]. With disease progression, nodules increasingly evolve to cavitation, then become cysts. Cysts usually persist and enlarge over time, being the predominant lesion in progressive cases [11,12]. In patients with suggestive clinical manifestations, typical HRCT findings are often sufficient to establish the diagnosis. When the HRCT is nondiagnostic, histological examination and IHC confirmation of antigens CD1a, S100 protein, or CD207, corroborated with imagistic findings, are hallmarks of a positive diagnostic [13]. PLCH is one of the few diseases in which BAL could still have a diagnostic value [14], specifically suitable for frail patients who cannot withstand surgical pulmonary biopsy [15]. The collected bronchial fluid can be used in fluid cytology and cell blocks [16,17]. In some cases, the use of molecular evaluations such as that of the *BRAF*-V600E mutation on the BAL cytology specimen and/or whole blood NGS for mutations and fusions in genes of the MAPK-ERK and related pathways provide supplementary diagnostic evidence, especially for atypical PLCH, and could stratify different pathological phenotypes [18]. The limitation specifically in our case was the fact that we did not perform an 18F-fluorodeoxyglucose positron emission tomography-based (18F-FDG PET/CT) imaging for staging as recommended by the new international expert consensus recommendations for the diagnosis and staging of adult LCH [18]. Unfortunately, 18F-FDG PET/CT was not provided in our hospital at the time of admission of the patient, and externalization for this imaging technic was not covered by the patient’s insurance. We present a longstanding ILD with a cystic appearance in an elderly non-smoker male with an atypical presentation on HRCT, who could not withstand a surgical lung biopsy, and where BAL disclosed characteristic findings of Langerhans cells associated with specific IHC markers providing a diagnosis of PLCH.

## Data Availability

The data that support the conclusions of this study could be available from the corresponding author, upon justified and reasonable request.
